# Evaluation of the Acute Hepatoprotective Potential of Hydroethanolic Extract of *Duranta erecta* L. Parts

**DOI:** 10.1155/2020/8815719

**Published:** 2020-12-09

**Authors:** Shadrack Donkor, Christopher Larbie, Gustav Komlaga, Benjamin Obukowho Emikpe

**Affiliations:** ^1^Applied Radiation Biology Centre, Radiological and Medical Sciences Research Institute, Ghana Atomic Energy Commission, Legon, Accra, Ghana; ^2^Department of Biochemistry and Biotechnology, Kwame Nkrumah University of Science and Technology (KNUST), Kumasi, Ghana; ^3^Department of Pharmacognosy, Kwame Nkrumah University of Science and Technology (KNUST), Kumasi, Ghana; ^4^Department of Pathobiology, School of Veterinary Medicine, Kwame Nkrumah University of Science and Technology (KNUST), Kumasi, Ghana

## Abstract

Liver disease is a major health problem and its treatment is costly in most developing countries with attendant adverse effects. This study aimed at determining the acute hepatoprotective efficacy of *Duranta erecta* hydroethanolic extracts of leaves, ripe and unripe fruits against CCl_4_-, and acetaminophen-induced hepatotoxicity in animals. *Materials and Methods*. CCl_4_ (1 mL/kg body weight in olive oil) and acetaminophen (500 mg/kg b.wt) were used to induce hepatotoxicity in the animals. Animals were treated with extracts at 250 mg/kg b.wt and standard drug, silymarin (100 mg/kg), for 7 days. Hepatoprotective efficacy was assessed by assaying serum biochemical markers such as alanine aminotransferase (ALT), aspartate aminotransferase (AST), alkaline phosphatase (ALP), gamma-glutamyl transferase (*γ*GT), bilirubin (Bil), antioxidative biomarkers including reduced glutathione (GSH), glutathione peroxidase (GPx), glutathione transferase (GST), superoxide dismutase (SOD), malondialdehyde (MDA), hydrogen peroxidase (H_2_0_2_), and nitric oxide (NO), as well as histological observations. *Results*. Exposure of the animals to CCl_4_ and acetaminophen resulted in liver injury as evidenced by elevated ALT, AST, ALP, *γ*GT, Bil, MDA, H_2_O_2_, and NO levels with resultant derangement in liver microarchitecture. Pretreatment with hydroethanolic extracts, particularly ripe fruits of *Duranta erecta*, led to a reduction in these indicators and an increase in GSH, GPx, GST, and SOD levels. Biochemical data were supported by improvement in liver structure. *Conclusion*. The findings suggest that hydroethanolic *Duranta erecta* ripe fruits extract possesses hepatoprotective and antioxidative activities against CCl_4_- and acetaminophen-induced toxicity and could be developed as a potent agent for drug-induced liver diseases.

## 1. Introduction

The liver is the largest organ in the human body and plays a critical role in influencing many life processes. It regulates the upkeep of internal environment through its multiple functions. This makes the liver highly vulnerable to substances that may be harmful to its cells, leading to liver injury or damage. Liver diseases are still a major health problem worldwide with a mortality rate of 2 million deaths per year [1] and affect people of all ages throughout the world. In Africa, particularly Ghana, accurate statistics on cause-specific mortality is not readily available; thus, there is the possibility to underestimate liver disease as the main cause of most deaths. Several agents, namely, viruses, chemicals, and pollutants are thought to be responsible for liver diseases. Liver diseases can initiate a cascade of events, resulting in extrahepatic morbidities which may eventually culminate into a reduced quality of life and mortality. According to the latest WHO data, liver disease-related deaths in Ghana accounted for 3,560 (3.27%) of the total deaths; Ghana was ranked 17th in the world with regard to liver disease-related deaths [2]. Treatments for liver diseases are costly in most developing countries. Most of the orthodox or synthetic drugs used in the treatment of liver diseases tend to have adverse effects on other body tissues. There is a need to improve the outcomes for patients with liver diseases with the search for natural products that exhibit low or no adverse effect. This has led to a growing interest in the use of traditional herbal medicines that supposedly possess hepatoprotective activity. Numerous studies have been carried to look out for natural products that exhibit hepatoprotective effects with low adverse reactions [3–6].

*Duranta erecta* L. (family Verbenaceae) is native to the tropical forest. In Ghana, it is cultivated as an ornamental hedge because of its profuse display of colourful flowers. It has been shown to possess antimicrobial [7], antioxidant [8], antifungal [9], antiparasitic [10], and antiviral [11] activities. The species has previously been demonstrated to contain important phytochemicals, such as flavonoids, phenols, and tannins, which can be exploited in the fight against oxidative stress conditions [8]. *Duranta erecta* has been used to treat various ailments since antiquity. The fruits have been used in the treatment of malaria and abscesses. The whole plant has been used to treat pneumonia, infertility, and neurological disorders [12]. The pharmacological and medicinal properties of the plant can be attributed to its phytochemical constituents such as durantol, pectolinaringenin, repennoside, repenins, and scutellarein [13].

Common liver injury models employed to screen hepatoprotective drugs include the use of carbon tetrachloride (CCl_4_) and some analgesics such as acetaminophen (paracetamol). The injury produced by these toxicants is because of their reactive metabolites. CCl_4_ is bioactivated by cytochrome P-450, resulting in the formation of trichloromethyl radical and reactive oxygen species. Trichloromethyl radical reacts with oxygen to form the highly toxic reactive trichloromethyl peroxyl radical, which initiates lipid peroxidation, resulting in a chain of reaction which eventually leads to liver damage [14]. Similarly, the toxicity of acetaminophen is due to a highly reactive metabolite N-acetyl-*p*-benzoquinoneimine, which is activated by cytochrome P-450, leading to oxidation of macromolecules such as lipid and protein [15].

Several pharmacological evaluations of *Duranta erecta* have been focused on its use as an antidiuretic, antitumour, antibacterial, and anthelmintic agent [16]. Little is known about its hepatoprotective property. The present study was aimed at assessing the protective effect of *Duranta erecta* hydroethanolic extracts (DRE) of the leaves, ripe fruits, and unripe fruits on carbon tetrachloride (CCl_4_)- and acetaminophen (Para))-induced liver damage in rats using biochemical and histological parameters.

## 2. Materials and Methods

### 2.1. Chemicals

Biochemical kits for the determination of alanine aminotransferase (ALT), alkaline phosphatase (ALP), gamma-glutamyl transferase (*γ*GT), total protein, albumin, bilirubin (total and direct), cholesterol, triglycerides, urea, and creatinine were obtained from Elitech, France. Silymarin, carbon tetrachloride (CCl_4_), acetaminophen, epinephrine, 5,5-dithiobis-2-nitrobenzoic acid (DTNB), hydrogen peroxide, reduced glutathione (GSH), sodium azide, ammonium ferrous sulphate, sorbitol, xylenol orange, and thiobarbituric acid (TBA) were purchased from SIGMA (USA). All other reagents were of analytical grade and were obtained from the British Drug Houses (Poole, UK).

### 2.2. Plant Collection and Extraction

Plant materials (leaves, ripe fruits, and unripe fruits) were collected in October 2017 within the vicinity of the Department of Biochemistry and Biotechnology Annex office, KNUST, and authenticated with voucher number KNUST/HM1/2017/L011 by Dr George Sam at the Department of Herbal Medicine, Faculty of Pharmaceutical Sciences, KNUST. The plant parts were treated and extracted as described previously [8]. Briefly, 60 g of pulverized leaf and fruit were extracted with 50% hydroethanol for 48 hours to obtain respective crude extracts; *Duranta erecta* leaves (DRL), *Duranta erecta* ripe (DRR), and unripe fruits extract (DRU).

### 2.3. Animals

Male albino rats (120–150 g) obtained from the animal facility of the Department of Biochemistry and Biotechnology, KNUST, were used in the study. They were kept under standard laboratory conditions and handled as stipulated in the guidelines of the Committee for the Purpose of Control and Supervision of Experiment on Animals (CPCSEA, New Delhi, India). Animals were housed in aluminium cages, suitably bedded with wood shaving. They were maintained under standard conditions of temperature and humidity and had free access to standard feed and water except for an overnight fast before being sacrificed. All animals were humanely handled during the experiment. In the experimental group of the animals, their body weight was taken into consideration to achieve approximately equal conditions among the groups. Animals were humanely handled according to the Guide for Care and Use of Laboratory Animals [17] and protocol for the animal study was reviewed and approved by a veterinarian on the team.

### 2.4. Treatments

Forty-four animals (eleven groups, *n* = 4) were used to study the acute hepatoprotective effect of *Duranta erecta* extracts on CCl_4_ and acetaminophen-induced liver damage. Details of experimental design and treatments are shown in Table 1. Group 1 served as vehicle control in both cases, received 1 mL/kg body weight (b.wt) of distilled water throughout the experiment. Groups II–VI were treated with 1 mL/kg b.wt CCl_4_ diluted with olive oil (1 : 1 vol/vol) for two successive days, the 2nd to 3rd day by intraperitoneal (i.p.) injection. Group II animals were maintained as CCl_4_ control without any drug treatment. Group III received standard hepatoprotective agent (100 mg/kg b.wt silymarin orally), from 1st to 7th day. Groups IV, V, and VI were pretreated with 250 mg/kg DRR, DRU, and DRL, respectively, by an oral route, from 1st to 7th day.

Similarly, in the Para-induced group, animals in groups VII–XI received acetaminophen (500 mg/kg b.wt) suspended in an appropriate volume of distilled water once daily, from 2nd to 7th day. Group VII was maintained on acetaminophen control without drug treatment. Groups IX to XI were pretreated with 250 mg/kg DRR, DRU, and DRL, respectively, for seven days, while Group XII was treated with 100 mg/kg silymarin orally, from 1st to 7th day. The rats were maintained on a normal diet and water *ad libitum*. A safe dose of 250 mg/kg body weight was used based on the safety assessment of leaves [18].

### 2.5. Blood Collection and Assessment of Hepatoprotective Activity

At the end of the experimental period, all the animals were sacrificed after an overnight fast on the 7th day by cervical dislocation. Blood samples were collected into gel-activated tubes via an incision in the cervical region with the aid of a sterile blade for biochemical assays. The following biochemical parameters, alanine aminotransferase (ALT), aspartate aminotransferase (AST), alkaline phosphatase (ALP), gamma-glutamyl transferase (*γ*GT), bilirubin (total and direct), total protein, albumin, total cholesterol, triglycerides, urea, and creatinine, were determined using the Selectra E (Vital Scientific, Japan) autoanalyzer.

### 2.6. Determination of Relative Organ Weight (ROW)

Excised liver of rats was washed in buffered normal saline and weighed to obtain the absolute liver weights (ALW). The relative organ weights (RLW) were calculated using the following formula:

(1) $$\begin{array}{c} \text{Relative liver weight weight}=\;\frac{\text{Absolute liver weight}}{\text{Body weight at sacrifice}}\times 100. \end{array}$$

### 2.7. Effect of Treatment of Oxidative Stress Parameters

Liver tissues (1 g) were homogenised in 10 mL of 100 mM KH_2_PO_4_ buffer containing 1 mM EDTA, pH 7.4, and centrifuged at 12,000 rpm for 30 min at 4°C. The supernatant was collected and used for the determination of nitric oxide (NO) [19], hydrogen peroxide (H_2_O_2_) [20], malondialdehyde (MDA) [21], reduced glutathione (GSH) [22], superoxide dismutase (SOD) [23,24], and glutathione peroxidase (GPx) [25].

### 2.8. Histology

Liver sections of the right lobes were preserved in 10% buffered formalin for fixation. Sections of 5–6 *μ*m in thickness were made and stained with haematoxylin and eosin for histological examination by pathologists.

### 2.9. Statistical Analysis

Results were analysed using GraphPad Prism 8.0 (GraphPad Software Inc., USA) for Windows. Experimental values were expressed as mean ± standard error of the mean (SEM). Data were assessed by ANOVA followed by Tukey's multiple comparison test to evaluate the significance between the various group. A *p*-value ≤0.05 was considered as significant.

Percentage protection was calculated based on the principal indicators of liver protection (AST, ALT, ALP, *γ*GT, TBIL, DBIL, and IBIL) using the following formula:

(2) $$\begin{array}{c} \text{\% protection}=\frac{\text{Value of toxin control}-\text{Value of test sample}}{\text{Value of toxin control}-\text{Value of normal control}}\times 100\text{.} \end{array}$$

## 3. Results

### 3.1. Effect of Treatment on Absolute and Relative Liver Weights

**Figure 1:**
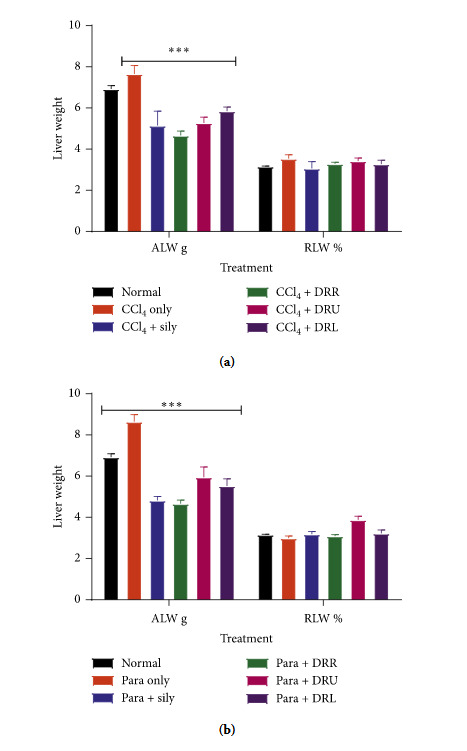
Effects of silymarin, DRR, DRU, and DRL on the absolute and relative liver weight of animals. ^*∗∗∗*^*P* < 0.001 from normal.

The intoxication with CCl_4_ and acetaminophen resulted in significant increases in the absolute liver weights (*P* < 0.001). Treatment with silymarin, DRR, DRU, and DRL reversed the trend (Figure 1). No significant changes were observed in the relative liver weights.

### 3.2. Effect of Treatment on Biochemical Parameters

The administration of CCl_4_ and acetaminophen caused significant increases (*p* < 0.05–0.01) in ALT, AST, ALP, *γ*GT, and TBil compared with the normal. Pretreatment with silymarin and extracts of *D. erecta* parts resulted in decreases in these parameters to near-normal levels when compared with CCl_4_ and Para only treated groups (Table 2).

### 3.3. Percentage Protection

**Figure 2:**
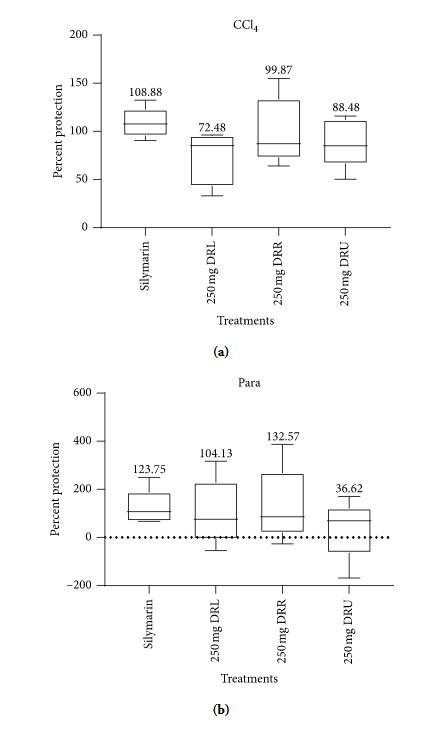
Percentage protection of silymarin and the extracts of *D. erecta* against CCl_4_ and acetaminophen.

The percentage protection of 100 mg/kg silymarin, DRL, DRR, and DRU at 250 mg/kg against CCl_4_ and acetaminophen is as shown in Figure 2. This was calculated as a function of principal indicators of liver function including ALT, AST, ALP, *γ*GT, and TBil. Silymarin and DRR at 250 mg/kg b.wt exhibited the highest percent protection against CCl_4_ and Para. Thus, the liver samples of these groups were subjected to histological, enzymatic, and nonenzymatic pro- and antioxidant assays. All extracts, however, offered some level of liver protection.

### 3.4. Effect of Treatment on the Antioxidant Defence System

The effect of treatments on oxidative stress parameters was assessed on silymarin- and DRR-treated groups since they showed the highest protection. Administration of CCl_4_ caused significant increases (*p* < 0.001) in H_2_O_2_, MDA, and NO levels, and a decrease in the GPx level compared with normal control while Para-only treatment resulted in similar observations except for NO levels. DRR cotreated with CCl_4_ resulted in decreases in H_2_O_2_ and MDA levels and increases in GPx, SOD, and NO compared with CCl_4_ only and Para only control groups. Silymarin cotreatments resulted in decreases in H_2_O_2_ and MDA levels with increases in GPx and SOD against Para while decreases in NO and MDA levels and increases in GPx and SOD were observed against CCl_4_ only control group (Table 3).

### 3.5. Effect of Treatment on Liver Histology

**Figure 3:**
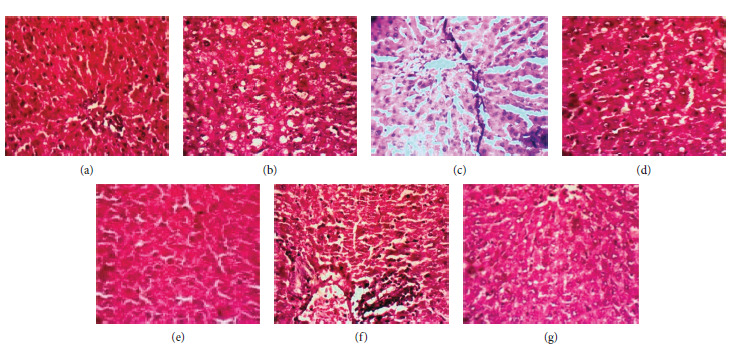
Photomicrographs showing liver of rats. (a) Normal-the hepatocytes are normal morphologically and arranged in cords around blood vessels except that the limiting plate appears shrunken, (b) CCl_4_ only-centrilobular coagulation necrosis of hepatocytes, (c) CCl_4_ + Sily-moderate centrilobular hepatocellular vascular degeneration, restricted more to the zone 3 hepatocytes and a few regenerative hepatocytes, (d) CCl_4_ +  DRR-diffuse hepatocellular atrophy, spotty hepatocellular necrosis, Kupffer cell hyperplasia, and moderate perivascular infiltrates in the portal tract, (e) Para only: Moderate centrilobular hepatocellular and cord atrophy, (f) Para + Sily: normal (no observable lesion), and (g) Para + DRR : LIVER- normal (no observable lesion).

The histological observations of the normal group revealed no observable lesions (Figure 3(a)). However, animals treated with CCl_4_ only showed hepatocellular atrophy and coagulation necrosis of hepatocytes with some inflammatory cells (Figure 3(b) ). Paracetamol-only treatment resulted in the development of moderate centrilobular hepatocellular and cord atrophy (Figure 3(e)). Pretreatment with silymarin (Figures 3(c) and 3(f)) showed no observable lesions to moderate centrilobular hepatocellular vascular degeneration and a few regenerative hepatocytes. Cotreatment with DRR (Figures 3(d) and 3(g)) attenuated the changes associated with CCl_4_- and Para-induced liver damage (Figure 3) with no observable damage to moderate diffuse hepatocellular atrophy and perivascular infiltrates in the portal tract.  Table 4 shows the lesion scores for the liver of normal and treated groups.

## 4. Discussion

Liver diseases are serious ailments claiming the lives of more than 2 million people globally in a year [26]. Despite recent advances in orthodox medicine, effective hepatoprotective medicine with low or no side effects is not easily available. Exposure of liver cells to toxicants is one of the ways to develop hepatoprotective drugs. In this present study, the effect of hydroethanolic extract of *Duranta erecta* was evaluated against CCl_4_- and acetaminophen-induced liver damage. These hepatotoxicants exert their deliberating effects through their active metabolites.

Changes in organ weight have been used as good indicators to evaluate the toxic effect of the test substance [27]. Alterations in organ weight may not directly reflect its functional state. Organ toxicity happens when there are changes in cellular structure or functions even after administration and elimination of the causative agent [28]. The observed significant increase in absolute liver weight after the administration of CCl_4_ and acetaminophen may be the result of direct toxicity of these hepatotoxicants and/or indirect toxicity leading to hypertrophy and liver damage (Figure 1). Pretreatment of animals with silymarin, DRR, DRU, and DRL decreased the absolute liver weights of treated animals compared to the hepatotoxin only group, indicating the ameliorative effect of the extracts.

Estimation of the activity of liver enzymes, namely, AST, ALT, ALP, and GGT in the event of hepatic injury is one of the useful tools in the study of hepatotoxicity as it can unfold the extent and type of damage [29, 30]. Serum AST and ALT are the cardinal indicators of liver injury, although ALT is a more selectively liver parenchymal enzyme than AST, whereas ALP is often used as an indicator of liver and gallbladder diseases [31]. Elevated serum GGT level has been associated with liver diseases and nonhepatic diseases, including chronic obstructive pulmonary disease. In CCl_4_- and acetaminophen-induced hepatotoxicity, the levels of liver enzymes in the bloodstream were affected and these reflect the status of liver function. The increased activities of these enzymes following the administration of the hepatotoxicants may be interpreted as hepatocyte damage and alterations in the membrane permeability. These findings are in agreement with previous studies [4, 6]. Pretreatment with the extracts and silymarin resulted in decreases of these parameters to near-normal levels in both CC_4_- and acetaminophen-induced damage, suggesting that extracts are beneficial in attenuating liver injury.

Bilirubin is a by-product of the breakdown of red blood cells in the liver. Accumulation, binding, conjugation, and excretion of bilirubin are related to the integrity and function of the liver. The increased serum total bilirubin level in the CCl_4_- and acetaminophen-treated rats observed in this study may be attributed to the failed processing capacity of the hepatocytes [32] which is a common feature of liver injury. However, the current results indicated that cotreatment with extracts were more effective in reversing the anomalies observed. Serum total protein level may indicate the state and type of damage to the liver [33]. Severe liver damage usually leads to decreased production of various proteins, resulting in reduced serum levels of total protein, albumin, and globulins. However, in the current study, no significant changes were observed due to the acute nature of the study.

Comparatively, the hepatoprotective potentials of DRR, DRU, DRL, and silymarin against CCl_4_ and acetaminophen showed that 250 mg/kg b.wt of DRR offered a better protective effect against CCl_4_- and paracetamol-induced toxicity after silymarin (Figure 2). The high protective effect of DRR and DRL could be attributed to the presence of phenols in the extracts as well as the free radical scavenging effect [8] which can deplete free radicals generated by the metabolism of CCl_4_ and acetaminophen.

Creatinine is a breakdown product in the muscle by creatine phosphate metabolism. Urea is the main product of protein catabolism. Creatinine and urea are important biochemical parameters for the diagnosis of renal impairment. Although CCl_4_ and Para treatments only did not result in elevations in these parameters, DRL, DRR, and DRU cotreatments resulted in significant decreases, signifying a possible nephroprotective potential attributable to the antioxidant and free radical scavenging properties of the extracts [8].

Free radicals produced by acetaminophen and CCl_4_ exert their deleterious effect on the liver through inflammatory response and oxidative stress, leading to initiation and progression of hepatic damage [35]. The mechanism of CCl_4_ injury results from its bioactivation to trichloromethyl peroxyl radical in the presence of oxygen by cytochrome P-450 enzymes, leading to peroxidation of lipids, covalent binding of macromolecules, disruption of metabolic mechanisms in mitochondria, inhibition of calcium pumps of microsomes, and secondary damage from an inflammatory process [36, 37]. The toxicity of acetaminophen is attributed to its highly reactive metabolite N-acetyl-*p*-benzoquinoneimine (NAPQI) which is activated by cytochrome P-450. NAPQI reacts with glutathione (GSH) causing its depletion to form protein adducts. This leads to mitochondrial dysfunction with its associated ATP depletion and oxidation stress which are thought to be critical for liver damage [34, 38]. Nature has an effective mechanism to prevent and neutralize the free radical-induced damage by employing an array of nonenzymatic and enzymatic reaction pathways.

MDA is a direct biochemical indicator for oxidative stress, and its elevation is an indication of an increased lipid peroxidation due to the production of superoxide, peroxyl, and hydroxyl radicals [39]. Increased MDA level, as evidenced in CCl_4_ and acetaminophen treated groups, is suggestive of enhanced lipid peroxidation and failure of antioxidant defence mechanisms to prevent the formation of excessive free radicals. The administration of the toxicants results in their active metabolites binding with cell protein, leading to lipid peroxidation of the cell membrane and endoplasmic reticulum, which in turn gives products like MDA. Current results suggested that the treatment with DRR caused a significant decrease in the levels of MDA through attenuation of lipid peroxidation and decreased production of free radical derivatives. This result indicates the hepatoprotective efficacy of the extract. This could be attributed to the antioxidant effect of the extract scavenging the free radicals, supplying a competitive substrate for unsaturated lipids in the membrane, and/or accelerating the repair mechanism of the damaged cell membrane [40].

H_2_O_2_ is produced in cells by a dismutation of superoxide radicals generated in the oxidative process by SOD [41]. It produces cytotoxicity in the endothelial cells of different organs [42]. Significantly elevated levels of H_2_O_2_ generation observed in acetaminophen- and CCl_4_-administered rats (Table 3) indicate the excessive formation of free radicals, resulting in hepatic damage. Pretreatment with DRR reversed these changes through the attenuation of lipid peroxidation and decreased the production of free radical derivatives as corroborated by a decreased level of MDA.

Nitric oxide (NO) is a vasodilator produced by endothelial nitrous oxide (NOS) which plays a critical role in the regulation of vascular tone by several mechanisms [43]. Low level of NO elicits hypertension, whereas excessive production could also precipitate hypotension. NO reacts with the superoxide radical to form the more potent oxidant peroxynitrite which is a highly toxic species and reacts with GSH, lipids, proteins, and DNA. The administration of CCl_4_ resulted in a significant increase (*p* < 0.001) of NO level, an indication of nitrosative stress. Pretreatment with the DRR reversed the change through attenuation of reactive nitrogen species. The ability of the extract to down regulate the level of radicals is an indication of its hepatoprotective protentials.

The body employs a set of endogenous antioxidant enzymes, such as SOD, GPx, and GST, as a defence mechanism to neutralise free radical-induced damage. SOD is a group of metalloenzymes which protects cells from the toxic effects of the endogenously generated superoxide radicals [44]. It is the first line of defence against free radical generation. SOD dismutates superoxide anion radical to H_2_O_2_ which can be rapidly converted to H_2_O and O_2_ by GPx [45]. GST is responsible for the detoxification of xenobiotics and takes part in maintaining glutathione homeostasis [24]. GSH is a multifunctional thiol-containing intracellular, nonenzymatic antioxidant. It is involved in the protection of the thiol group of proteins from oxidation by free radicals [46] and it acts as a substrate for GPx and GST. GPx and GST as antioxidant enzymes work together with GSH to decompose of H_2_O_2_ and other organic hydroperoxides [47]. Results of current studies indicated that the intoxication of the rats with the CCl_4_ significantly reduced the GPx level, compared with the control (Table 3) which was in line with other findings [48]. The decreased level of GPx and other antioxidant enzymes is most likely due to the overproduction of free radicals through oxidative stress that overwhelmed the antioxidant defence system. Pretreatment with DRR reversed these changes, resulting in upregulation in the activity of antioxidant enzyme and GPx which may suggest the development of an adaptive response to rid the body of oxidative stress. The observed modulation of oxidative stress by the hydroethanolic extracts of plant parts can be attributed to the reported in vitro antioxidant activities reported for Ghanaian [8], Nigerian [49], and Egyptian [50] cultivars. Furthermore, observations of the current study lay credence to the medicinal properties of ornament plants including hepatoprotection as previously reported for *Tecoma stans* L. [51] and other species.

The histological observation of the liver sections revealed hepatocellular atrophy and coagulation necrosis of hepatocytes with some inflammatory cells in the CCl_4_-induced damage whilst moderate centrilobular hepatocellular and cord atrophy was recorded in acetaminophen-induced rats. This could be due to the formation of the highly reactive metabolites of the various hepatotoxicants. The administration of DRR and silymarin resulted in no observable lesion in the acetaminophen-induced group which was close to normal cellular architecture and diffused vacuolar degeneration, Kupffer cell hyperplasia, and moderate perivascular infiltrates in the portal tract in the CCl_4_-induced rats (Figure 3, Table 4). These results are consistent with the various biochemical parameters that proved the hepatoprotective efficacy of the plant.

## 5. Conclusion

Based on the results obtained in this study, it is concluded that the extracts of *Duranta erecta* particularly the leaves and ripe fruits exhibit a strong protective effect against CCl_4_- and acetaminophen-induced liver injury by modulating serum and liver biochemical parameters, supported by histological observation. Thus, 50% hydroethanolic ripped fruit extract of *D. erecta* is hepatoprotective by inhibition of oxidative stress and improvement in liver microarchitecture and function attributable to its phytochemical constituents and can be exploited as a therapeutic agent against acute liver diseases.

## Figures and Tables

**Table 1:** Grouping of animals for the study.

Group number	Group name	Treatment
I	Normal	Animals orally received 1 mL/kg b.wt distilled water daily for 7 days
II	CCl_4_ only	Animals received 1 mL/kg b.wt CCl4 dissolved in olive oil (1 : 1 v/v) i.p. on 2nd and 3rd days
III	Sily + CCl_4_	Animals orally received 100 mg/kg b.wt silymarin dissolved in distilled water daily for 7 days and 1 mL/kg b.wt CCl_4_ on 2nd and 3rd days
IV	DRR + CCl_4_	Animals received 250 mg/kg b.wt DRR for 7 days and 1 mL/kg b.wt CCl_4_ on 2nd and 3rd days
V	DRU + CCl_4_	Animals received 250 mg/kg b.wt DRU for 7 days and 1 mL/kg b.wt CCl_4_ on 2nd and 3rd days
VI	DRL + CCl_4_	Animals received 250 mg/kg b.wt DRL for 7 days and 1 mL/kg b.wt CCl_4_ on 2nd and 3rd days
VII	Para only	Animals received 500 mg/kg b.wt Para (i. p.) daily for 7 days
VIII	Sily + Para	Animals orally received 100 mg/kg b.wt silymarin dissolved in distilled water and 500 mg/kg b.wt Para (i. p.)
IX	DRR + Para	Animals received 250 mg/kg b.wt DRR for 7 days and 500 mg/kg b.wt Para (i. p.)
X	DRU + Para	Animals received 250 mg/kg b.wt DRU for 7 days and 500 mg/kg b.wt Para (i. p.)
XI	DRL + Para	Animals received 250 mg/kg b.wt DRL for 7 days and 500 mg/kg b.wt Para (i. p.)

**Table 2:** Effect of treatment on biochemical markers of animals in CCl4 and Para treatments.

	Normal	CCl_4_ only	Sily + CCl_4_	DRL + CCl_4_	DRR + CCl_4_	DRU + CCl_4_	Para only	Sily + Para	DRL + para	DRR + Para	DRU + Para
Total protein (g/L)	64.35 ± 1.63	76.47 ± 1.79	72.73 ± 3.91	65.00 ± 4.89	64.90 ± 1.75	65.35 ± 3.16	76.77 ± 2.97	72.48 ± 1.62	63.30 ± 5.89	62.15 ± 2.21	59.68 ± 2.76
ALB (g/L)	38.05 ± 1.41	38.80 ± 0.72	35.75 ± 1.24	32.00 ± 3.29	35.30 ± 1.12	35.40 ± 0.60	38.60 ± 2.46	38.88 ± 1.78	31.63 ± 2.43	36.85 ± 1.76	33.30 ± 1.26
GLO (g/L)	26.30 ± 1.97	37.67 ± 1.11	36.98 ± 5.05	33.00 ± 2.67	29.60 ± 1.75	29.95 ± 2.73	38.17 ± 5.40	33.60 ± 1.33	31.68 ± 4.48	25.30 ± 2.97	26.38 ± 2.08
ALT (U/L)	50.00 ± 2.31	178.60 ± 6.67^a^	39.95 ± 2.10^b^	57.55 ± 2.21^b^	66.50 ± 4.17^b^	69.20 ± 0.83^b^	151.33 ± 6.11^a^	42.13 ± 3.16^c^	74.78 ± 3.94^c^	82.78 ± 4.04^c^	80.80 ± 3.11^c^
AST (U/L)	127.13 ± 6.47	280.85 ± 25.96^a^	141.58 ± 8.08^b^	229.93 ± 4.77^ab^	182.28 ± 4.86^ab^	203.58 ± 15.36^ab^	348.90 ± 14.32^a^	194.18 ± 5.86^ac^	251.85 ± 13.11^ab^	157.80 ± 15.70^c^	256.35 ± 4.65^ab^
ALP (U/L)	459.85 ± 15.73	660.00 ± 25.64^a^	395.10 ± 16.85^ab^	552.48 ± 13.32^ab^	349.98 ± 30.79^ab^	444.23 ± 43.98^ab^	540.67 ± 18.26^a^	339.05 ± 26.17^ac^	284.70 ± 30.98^ac^	227.65 ± 24.00^ac^	402.8 8 ± 9.79^ac^
GGT (U/L)	10.90 ± 1.05	19.53 ± 0.41^a^	9.80 ± 0.38^b^	11.23 ± 1.53^b^	9.90 ± 1.15^b^	9.53 ± 2.14^b^	18.30 ± 0.69^a^	13.33 ± 0.85	8.03 ± 0.90^c^	7.33 ± 0.40^c^	13.15 ± 1.67
TBil (mmol/L)	1.67 ± 0.19	5.35 ± 0.20^a^	1.64 ± 0.12^b^	2.22 ± 0.15^b^	2.35 ± 0.36^b^	2.29 ± 0.13^b^	2.23 ± 0.11	1.54 ± 0.12	2.54 ± 0.18	2.38 ± 0.05	2.58 ± 0.25
DBil (mmol/L)	0.80 ± 0.13	1.04 ± 0.21	0.92 ± 0.09	1.19 ± 0.15	1.21 ± 0.18	0.98 ± 0.04	1.04 ± 0.25	0.73 ± 0.10	1.37 ± 0.14	1.25 ± 0.07	1.02 ± 0.33
IBil (mmol/L)	0.88 ± 0.16	4.31 ± 0.03^a^	0.73 ± 0.03	1.03 ± 0.17	1.13 ± 0.36	1.31 ± 0.09	1.20 ± 0.21	0.81 ± 0.10	1.17 ± 0.07	1.14 ± 0.11	2.16 ± 0.10^c^
Creat (*μ*mol/L)	27.68 ± 4.09	28.57 ± 4.06	35.95 ± 8.03	11.20 ± 3.83^ab^	20.90 ± 2.70	18.96 ± 2.41	27.10 ± 2.78	26.53 ± 2.45	19.38 ± 3.95	11.75 ± 1.67^ac^	19.73 ± 1.94
Urea	3.58 ± 0.22	9.89 ± 1.16^a^	8.30 ± 1.00	4.99 ± 0.52	6.86 ± 0.41	5.18 ± 0.53	6.60 ± 1.48	5.53 ± 0.87	3.74 ± 0.37	5.42 ± 0.70	5.15 ± 0.29
TChol (mmol/L)	2.53 ± 0.19	3.03 ± 0.43	3.29 ± 0.09	2.04 ± 0.17	2.44 ± 0.22	2.37 ± 0.20	3.24 ± 0.30	3.12 ± 0.13	2.01 ± 0.14	2.07 ± 0.14	1.49 ± 0.19
Trig (mmol/L)	1.67 ± 0.22	0.90 ± 0.09	0.90 ± 0.09	1.27 ± 0.30	0.62 ± 0.15	0.87 ± 0.13	1.62 ± 0.19	1.07 ± 0.13	1.48 ± 0.59	0.98 ± 0.26	0.87 ± 0.25
VLDL (mmol/L)	0.75 ± 0.09	0.40 ± 0.06	0.40 ± 0.04	0.58 ± 0.13	0.30 ± 0.07	0.40 ± 0.07	0.73 ± 0.09	0.50 ± 0.04	0.40 ± 0.12	0.68 ± 0.26	0.71 ± 0.20

Statistical significance: ^a^*p* < 0.05–0.001 from normal; ^b^*p* < 0.05–0.001 from CCl_4_ only; ^c^*p* < 0.05–0.001 from Para only.

**Table 3:** Effect of treatment on liver oxidative stress parameters.

	Normal	CCl_4_ only	Para only	CCl_4_ + Sily	Para + Sily	CCl_4_ + DRR	Para + DRR
H_2_O_2_ (*μ*mole/mg protein)	69.78 ± 6.74	99.06 ± 10.63^a^	122.31 ± 17.37^a^	110.17 ± 8.07^a^	56.13 ± 3.13^c^	65.58 ± 6.97^b^	121.63 ± 16.79^a^
MDA (*μ*mole/mg protein)	3.59 ± 0.68	14.25 ± 1.46^a^	14.82 ± 5.01^a^	3.25 ± 2.29^b^	3.01 ± 1.32^c^	3.84 ± 2.36^b^	3.94 ± 4.36^c^
GSH (*μ*mole/mg protein)	74.72 ± 2.34	72.44 ± 1.64	83.18 ± 6.07	78.52 ± 1.06	77.51 ± 2.70	78.34 ± 2.32	74.82 ± 2.27
GPx (*μ*mole/mg protein)	713.82 ± 48.18	563.03 ± 24.57^a^	682.95 ± 49.60	868.15 ± 71.57^b^	1225.42 ± 37.90^c^	906.05 ± 65.94^ab^	1055.99 ± 42.72^ac^
GST (µmole/min/mg protein)	1.07 ± 0.11	0.78 ± 0.12	1.22 ± 0.18	0.90 ± 0.19	0.91 ± 0.22	2.14 ± 1.0	2.63 ± 0.68
SOD (Units/mg protein)	25.28 ± 1.70	19.94 ± 0.84	25.83 ± 2.38	31.75 ± 2.48^b^	43.88 ± 1.31^ac^	32.60 ± 2.47^b^	37.51 ± 1.05^ac^
Nitric oxide (*μ*mole/L)	0.98 ± 0.05	2.77 ± 0.47^a^	0.98 ± 0.03	1.19 ± 0.16^b^	0.49 ± 0.02	2.14 ± 0.16^a^	2.53 ± 0.17^ac^

Statistical Significance: ^a^*p* < 0.05–0.001 from normal; ^b^*p* < 0.05–0.001 from CCl_4_ only; ^c^*p* < 0.05–0.001 from Para only.

**Table 4:** Scoring of lesions.

Group	Score	Lesion
Normal	0	No observable lesion
CCl_4_	4	Hepatocellular atrophy and coagulation necrosis of hepatocytes with some inflammatory cells
Para only	2	Moderate centrilobular hepatocellular and cord atrophy
CCl_4_ + Sily	2	Moderate centrilobular hepatocellular degeneration and a few regenerative hepatocytes
Para + Sily	0	No observable lesion
DRR + CCl_4_	3	Diffused vacuolar degeneration, Kupffer cell hyperplasia, moderate perivascular infiltrates in portal tract
DRR + Para	0	No observable lesion

## Data Availability

The data used and/or analysed during the current study are available from the corresponding author upon request.
